# Muscular and functional effects of partitioning exercising muscle mass in patients with chronic obstructive pulmonary disease - a study protocol for a randomized controlled trial

**DOI:** 10.1186/s13063-015-0698-x

**Published:** 2015-04-27

**Authors:** Andrè Nyberg, Didier Saey, Mickaël Martin, François Maltais

**Affiliations:** Centre de recherche de l’Institut universitaire de cardiologie et de pneumologie de Québec, 2725, chemin Sainte-Foy, Québec, G1V 4G5 Canada; Département de réadaptation, Faculté de médecine, Université Laval, 1050, avenue de la Médecine, Québec, G1V 0A6 Canada; Département de médecine, Faculté de médecine, Université Laval, 1050, avenue de la Médecine, Québec, G1V0A6 Canada

**Keywords:** COPD, Elastic resistance, Exercise capacity, Dyspnea, High-repetitive, Mechanisms, Quality of life, Randomized controlled trial

## Abstract

**Background:**

Low-load, high-repetitive single-limb resistance training may increase limb muscle function and functional exercise capacity in patients with chronic obstructive pulmonary disease (COPD) while minimizing the occurrence of limiting exertional symptoms. Whether high-repetitive single-limb resistance training would perform better than high-repetitive two-limb resistance training is unknown. In addition, the mechanisms underlying possible benefits of high-repetitive resistance training has not been investigated. The aims of this study are to compare single versus two-limb high-repetitive resistance training in patients with COPD and to investigate mechanisms of action of these training modalities.

**Methods/Design:**

This trial is a prospective, assessor-blind, randomized controlled trial. The participants are patients with stable severe to very severe COPD who are older than 40 years of age and healthy controls. The intervention is single-limb, high-repetitive, resistance training with elastic bands, three times/week for 8 weeks.

The control is two-limb high-repetitive resistance training with elastic bands, three times/week for 8 weeks.

The primary outcomes is change in the 6-min walking distance after 8 weeks of single-limb or two-limb high-repetitive resistance training.

The secondary outcomes are changes in limb muscle strength and endurance capacity, key protein involved in quadriceps anabolic/catabolic signalization, fiber-type distribution and capillarization, subjective dyspnea and muscle fatigue, muscle oxygenation, cardiorespiratory demand and health-related quality-of-life after 8 weeks of single-limb or two-limb high-repetitive resistance training.

The acute effects of single-limb versus two-limb high-repetitive resistance training on contractile fatigue, exercise stimulus (the product of number of repetition and load), subjective dyspnea and muscle fatigue, muscle oxygenation, and cardiorespiratory demand during upper and lower limb exercises will also be investigated in patients with COPD and healthy controls.

Randomization will be performed using a random number generator by a person independent of the recruitment process, using 1:1 allocation to the intervention and the control group using random block sizes.

Blinding: All outcome assessors will be blinded to group assignment.

**Discussion:**

The results of this project will provide important information to help developing and implementing customized exercise training programs for patients with COPD.

**Trial registration:**

ClinicalTrials.gov Identifier NCT02283580 Registration date: 4 November 2014. First participant randomized: 10 November 2014.

**Electronic supplementary material:**

The online version of this article (doi:10.1186/s13063-015-0698-x) contains supplementary material, which is available to authorized users.

## Background

The key disabling features in chronic obstructive pulmonary disease (COPD) include exercise intolerance, dyspnea and leg fatigue [1-3]. Increased dyspnea during physical activity is also a major reason for patients seeking medical care and is associated with lower quality of life [4]. Limb muscle dysfunction is another frequent manifestation of COPD. Decreased limb muscle strength and endurance have been documented in this disease [5,6]. Since limb muscle dysfunction in COPD is strongly associated with exercise intolerance, improving muscle function is of upmost importance. Limb muscle weakness is considered as a significant determinant of reduced exercise capacity in COPD [6] and is present in all stages of the disease [7]. As a consequence, most previous studies on resistance training in patients with COPD primarily focus on increasing muscle strength, typically using high loads and low number of repetitions [6,8]. However, the benefits of this resistance training strategy on functional capacity (for example, walking) are small in this group of patients [8]. Since quadriceps endurance is a determinant of physical activity level [5] and may be reduced to a larger extent than quadriceps strength [9,5], the possibility of improving limb muscle endurance is of interest. To achieve this goal, high-repetition resistance exercises is recommended [10]. Furthermore, regardless the type of exercise performed, achieving an optimal exercise stimulus for involved muscles could be challenging in patients with COPD because ventilatory limitation and dyspnea may occur prior to contracting muscles being maximally stressed [11,12]. This is of importance because the degree of exercise stimulus is central to optimize benefits from the exercise training performed.

Partitioning the exercising muscle mass (that is, exercising using a single limb at a time) is a novel strategy used to reduce the extent to which the ventilatory limitation hinders the ability to reach sufficient exercise stimulus and to optimize the effects of the exercise training in patients with COPD [13]. A randomized controlled study using the concept of partitioning the exercising muscle mass performed as high-repetitive single-limb resistance training have recently been conducted by our group [14]. All resistance exercises within this study were executed with a single limb at a time in order to minimize to load on the ventilatory system to maximize the muscle-specific exercise stimulus [15,14]. It was found that 8 weeks of high-repetitive single-limb resistance training can improve walking capacity and muscle function while avoiding the occurrence of limiting exertional symptoms in patients with moderate to severe COPD [14]. However, since there was no active comparator, we do not know whether the single-limb execution of the resistance exercises would be more beneficial than when involving larger amount of muscle mass [16,17,14]. It could be anticipated that partitioning the exercising muscle mass during high-repetitive resistance training would allow the patients to maintain a high number of repetitions while being able to use higher loads or higher local training intensities than if a larger amount of muscle mass would be involved (for example, exercising with both legs or both arms simultaneously).

Another limitation of our study on high-repetitive single-limb resistance training is that potential structural and muscle adaptations could not be confirmed as the mechanisms underlying the improvements were not assessed [14]. The impact of high-repetitive resistance training on muscle fatigue was also not explored. This is potentially important considering that unpublished data from our laboratory indicate that high-repetitive single-leg exercise may induce muscle fatigue in the absence of muscle deoxygenation, considering that larger training effects have been shown in patients with COPD who develop quadriceps contractile fatigue during exercise training [18].

Lastly, the majority of patients included in this previous research had moderate airflow limitation, whereas high-repetitive single-limb resistance training would appear particularly suited for patients with advanced ventilator limitation. Thus, the overall aim of the current project is to investigate the muscular and functional effects of high-repetitive resistance training, in patients with severe to very severe COPD and to better understand the mechanisms of action of this training modality.

## Methods/Design

**Figure 1:**
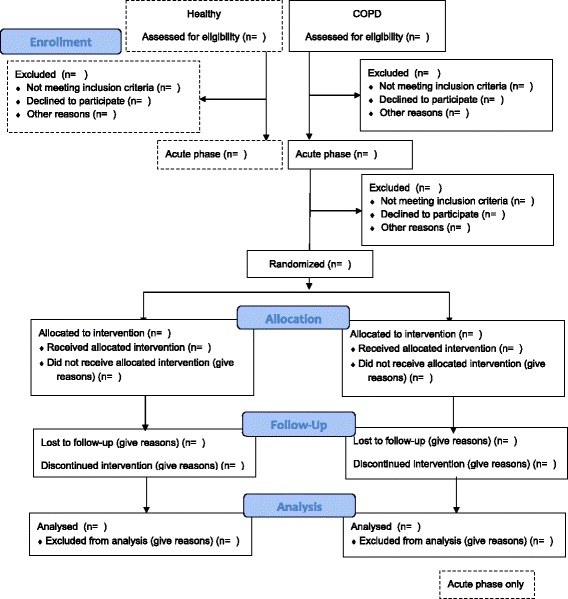
Consolidate standards of reporting trials (CONSORT) flow diagram of trial design.

The study is a prospective, assessor-blind, block randomized, controlled, parallel-group trial constructed in accordance to the consolidate standards of reporting trials guidelines [19,20] (Figure 1). The study contains a chronic and an acute phase. In the chronic phase, the benefits of 8 weeks of single-limb and two-limb high-repetitive resistance training will be compared in patients with severe and very severe COPD. The acute phase will be used to investigate, at baseline, the acute physiological effects of single-limb and two-limb high-repetitive resistance training in patients with COPD and also in healthy controls. An overview of the two phases including a description of the testing procedure can be seen in Table 1.

**Table 1:** **Acute and chronic phase description**

**Acute phase**			**Chronic Phase**		
**Patients with COPD & Healthy controls**			**Patients with COPD**		
Visit 1	Visit 2 and 3*	R	Training	Visit 4	Visit 5 and 6
		A			
● Anthropometrics	● Muscle biopsy or 6MWT	N	High-repetitive single limb elastic band resistance training	● Anthropometrics	● Muscle biopsy (one visit)
● Voorips questionnaire		D		● Voorips questionnaire	High-repetitive single limb or two limb elastic band exercises
● CAT questionnaire †	High-repetitive single limb or two limb elastic band exercises	O		● CAT questionnaire	
				● Isokinetic measurements	
● Pulmonary function		M		● UULEX	● Muscle oxygenation
● Isokinetic measurements		I		● 6MWT	● Cardiorespiratory response
● UULEX †	● Muscle oxygenation				● Exertional symptoms
● 6MWT †	● Cardiorespiratory response	Z			● Muscle fatigability
● 25 RM testing	● Exertional symptoms	A			● Exercise stimulus
	● Muscle fatigability	T	High-repetitive two limb elastic band resistance training		
	● Exercise stimulus	I			
		O			
		N			
**Baseline measurements**			**8 weeks training**	**Post assessment**	

*Visit order is randomized. All visits are sepearated by at least 48 h.

†Only performed by patients with COPD.

6MWT, 6-minute walk test; CAT, COPD Assessment Test; COPD, chronic obstructive pulmonary disease; RM, repetition maximum, UULEX, unsupported upper limb exercise test.

### Objectives

#### Primary outcome

The primary outcome is change in the 6-min walking distance after 8 weeks of single-limb versus two-limb high-repetitive resistance training.

#### Secondary outcomes

The secondary outcomes are changes in the following:

1. quadriceps endurance capacity;
2. key protein involved in quadriceps anabolic/catabolic signalization, fiber-type distribution, enzymatic activities and capillarization;
3. dyspnea and muscle fatigue perception;
4. quadriceps oxygenation;
5. cardiorespiratory response; and
6. health-related quality-of-life, after 8 weeks of single-limb versus two-limb high-repetitive resistance training.

Secondary outcomes will also include acute effects of single-limb versus two-limb high-repetitive resistance exercises on the following:

1. quadriceps contractile fatigue,
2. exercise stimulus,
3. dyspnea and muscle fatigue perception,
4. muscle oxygenation and cardiorespiratory demand, and
5. limb muscle function (maximal strength and endurance).

### Hypotheses

The following hypotheses are proposed:

1. Single-limb high-repetitive resistance training will provide a larger gain in the 6-min walking distance than the two-limb high-repetitive resistance training in patients with severe to very severe (stage 3 to 4) COPD.
2. Eight weeks of single limb high-repetitive resistance training will be associated with larger physiological (muscle endurance, fatigue and deoxygenation) and structural (muscle protein synthesis, fiber-type distribution and capillarization) adaptations to training, and lower cardiorespiratory demand, as well a greater increase in health-related quality-of-life in comparison to two-limb high-repetitive resistance training.
3. Two-limb high-repetitive resistance training will lead to larger restraints on the ventilatory system and higher dyspnea scores and, to reduced muscle fatigue, deoxygenation and localized exercise stimulus in comparison to single-limb high-repetitive resistance training in patients with COPD. Healthy controls will be able to train both legs/arms simultaneously without central constraints and with no differences in muscle fatigue or exercise stimulus between the two high-repetitive regimens.

### Settings

The study will be performed at Institut Universitaire de cardiologie et de pneumologie de Québec, Québec, Canada. Recruitment and data collection will begin in November 2014.

### Sample size and eligibility criteria

A total of 30 patients will be included. This sample size was calculated with the premises that 13 patients in each group would be required to obtain sufficient power to detect a 34-m (±30) between-group difference in the 6-minute walk test (6MWT), α = 0.05, β = 0.20 (80% power), and a two-tailed test of significance, based on the previous study on high-repetitive resistance training in COPD [14]. We are aiming to detect at least a 30-m difference between the two groups since this value is considered to be clinically significant in this population [21]. Considering a 15% drop-out rate in this type of study, a total of 30 patients will be recruited. In addition, for the acute phase of this protocol, a sample size of 11 patients with COPD and 11 controls is required to detect a relevant mean difference of 11% (±9) in limb muscle contractile fatigue between single-limb and two-limb execution of the exercises performed in the acute phase of this protocol for an α = 0.05, β = 0.20 (80% power), and a two-tailed test of significance based on a study by Rossman *et al*. comparing single-leg to two-leg knee extension in healthy adults [22].

### Inclusion criteria (chronic obstructive pulmonary disease)

The inclusion criteria for participation in the trial are as follows:

1. age ≥ 40 years,
2. cumulative (current or previous) smoking history > 10 pack-years, and
3. postbronchodilator FEV_1_ ≥ 50% predicted Global Initiative for Chronic Obstructive Lung Disease Spirometric stage 3 or 4 [4].

### Exclusion criteria

The exclusion criteria for participation in the trial are as follows:

1. recent COPD exacerbation (< 6 weeks),
2. neuromuscular and/or orthopedic disorders that compromise participation to an exercise program,
3. recent cancer,
4. unstable cardiac disease and cardiac stimulator,
5. asthma (current),
6. low body weight or obesity (body mass index < 20 kg/m^2^ or > 30 kg/m^2^),
7. significant hypoxemia at rest (SpO_2_ < 85%), or
8. a daily dose > 10 mg of systemic prednisone.

### Inclusion criteria (healthy controls)

The inclusion criteria for healthy controls are as follows:

1. age ≥ 40 years and
2. normal pulmonary function.

### Exclusion criteria (healthy controls)

The exclusion criteria for participation as a healthy control are as follows:

1. neuromuscular and/or orthopedic disorders compromising participation to an exercise program or
2. physically active (> 9) on the Voorips questionnaire [23].

### Description of intervention and control groups

Patients with COPD will be randomized to either single or two-limb high-repetitive resistance training. Identical high-repetitive resistance exercise regimen will be used for both interventions, with one exception; the intervention group will perform the exercise regimen using a single limb at a time while the control group will, depending on exercise, perform the training using either both arms or both legs. A total of three sets will be performed for each limb. Training time will be shorter for the two-limb high-repetitive training group in comparison to the single-limb training group. To minimize any confounding effects of different amount of time spent with research staff, the control group will perform a light intensity balance exercise that will compensate for the difference in training time between the two interventions.

Both interventions will be provided on the basis of three sessions per week for 8 weeks. They will be performed under direct supervision, individually or in small groups of 2 to 4 participants. The care provider supervising the training will receive standardized education before the start of intervention consisting of written, oral and visual instructions on how to perform and instruct the exercises used in both high-repetitive protocols.

Before the actual training, there will be a 5-minute warm-up period on a stationary cross-trainer, focusing on muscle groups that will be trained during various exercises, including the latissimus row (m. latissimus dorsi), chest press (m. pectoralis major and m. deltoid anterior), knee extension (m. quadriceps), shoulder flexion (m. deltoid anterior), leg curl (m. hamstrings), elbow flexion (m. biceps brachii) and plantar flexion (mm. triceps surae). All exercises will be performed with elastic bands (Thera-Bands, The Hygenic Corporation 1245 Home Ave. Akron, OH 44310) with the exception of plantar flexion that will be executed using a weight-machine (Sport Art Fitness, Woodinville, WA, USA). Extended description of the exercise programs for the single-limb and the two-limb group including detailed including illustrations of start and end positions as well as execution of exercise movement is explained more in detail in the additional files [see Additional file 1 and 2].

Unilateral or bilateral exercises with high repetitions and low amount of rest (one minute) are selected for this trial in line with recommendations from the ACSM [10]. Moderate to fast velocities is recommended when performing a larger number of repetitions, that is, at least 15 repetitions. Participants will be positioned so that the elastic band(s) are stretched 100% at end of movement for each exercise, respectively. Since the most clinically effective portion of elastic resistance is 25% to 250% elongation, all exercise start and stop positions are within this interval [24]. The relaxed length of the elastic resistance bands from insertion are standardized to 85 cm for each side, pre-stretched 20 times to stabilize the material [25].

Individual starting resistance will be set at 25 repetition maximum (25 RM) in the different exercises. The resistance for each exercise and the progression of resistance will be individually determined. Progression will be done separately for the different exercises in accordance to ACSM recommendations [10]. That is, a participant performing >30 repetitions will result in an increase in resistance. The criteria for progression should be fulfilled in two subsequent sessions or two out of three successive sessions. Resistance will be increased with approximately 10% by changing the tension of the resistance bands or adding weights to the weight machine [10].

Cool down/stretching will consist of four stretching exercises for involved muscle groups. Holding each stretch 30 seconds to mild discomfort, two sets for each limb (that is, two minutes of active stretching for each muscle), in accordance to American College of Sports Medicine recommendations [26].

### Procedure

Participants will perform three visits for the acute phase of the protocol. These visits are also used as baseline testing for patients with COPD who will contribute to the chronic phase of the protocol. The same evaluation will also be performed after the 8-week training program in patients with COPD. Pre- and post-tests will be performed at similar time of day, with the same rest between test occasions and between tests.

Since a subject’s motivation may determine attainment of maximal effort [5], both pre- and post-tests are performed using standardized information and encouragement [27]. No additional follow-up other than directly after end of intervention period is used within this trial. The perception of dyspnea and muscle fatigue will be measured with the modified Borg scale [28] before and directly after all physical tests as commonly and reliably used in patients with COPD [29,14,30].

#### Screening tests

During the first visit, all procedures will be explained and the informed consent form will be reviewed and signed. Participants will then fill the Voorips physical activity level questionnaire that is validated for elderly individuals [23] and applied to patients with COPD [31]. Participants will complete pulmonary function assessment (plethysmography, spirometry) while being on their usual bronchodilator therapy. Anthropometric measurements will include height, weight, body composition and thigh subcutaneous skinfold. The latter is relevant for measurement of muscle oxygenation. Body composition will be assessed by bioelectrical impedance (InBody520, Body Composition Analyzer, Biospace, Seoul, Korea).

### Outcome measures

#### Functional tests

The 6MWT will be used to assess the primary outcome, walking distance [32]. The walking course will be 30 meters in length, and the patients will be instructed in accordance to standardized guidelines [33] to walk as far as possible in 6 minutes. One practice test will be performed to minimize the risk of learning effect. The highest walking distance of the two 6-min walks will be chosen as baseline value.

To measure unsupported upper limb endurance capacity the unsupported upper limb exercise test (UULEX) will be used [34]. Participants will hold a plastic bar (0.2 kg), at shoulder width and will be asked to raise it from hip to the UULEX eight level chart for one minute at each level with a cadence of 30 movements per minute. If a patient reaches the highest level, the plastic bar will be replaced by a heavier one every minute. There are five different bar weights (0.2, 0.5, 1, 1.5, 2 Kg), and participants will continue on the highest level until symptom limitation.

#### Limb muscle function

Limb muscle function will be evaluated in two ways. Muscle strength and endurance will be evaluated during isokinetic knee extension and shoulder flexion using the Biodex Multi-Joint System 4 (Biodex Corp., Shirley, New York). Both knee extension and shoulder flexion will be performed unilaterally on the self-reported dominant side in accordance to manufacturer recommendations. In addition, knee extension will also be performed with both lower limbs in order to compare single to two-limb execution. Each movement will be performed 25 consecutive times using maximal effort during the concentric phase and relaxation during the eccentric phase. Two different aspects of muscle function will be measured. Peak torque from the highest contraction will be used for maximal strength and total work generated from all contractions will be used for endurance. The tests will be performed at an angular velocity of 60° · s^−1^, which is considered ideal for evaluating muscle strength in patients with COPD [35] and is found reliable in endurance testing procedures [36]. Before each test, participants will perform five submaximal contractions to familiarize themselves with the equipment and testing procedures, followed by 2 minutes of standardized rest [27]. Shoulder flexion will be performed between 0° to 90° with the elbow extended and the forearm pronated. Knee extension will be performed between 90° flexion to maximal individual extension minus 5° to lower the risk for a passive resistance from the hamstrings muscles. No visual feedback is given during tests. Standardized encouragement will be given.

Limb muscle function will also be evaluated, during visit two and three of the acute phase and during post assessment in the chronic phase, in the following six resistance exercises: latissimus row, chest press, knee extension, shoulder flexion, leg curl and elbow flexion [37-40]. All exercises will be executed using elastic resistance bands (Thera-Bands, The Hygenic Corporation, Akron, OH, USA) and will be performed seated using the chair of the Biodex Multi-Joint System 4 (Biodex Corp., Shirley, New York, USA). The positioning of the participants will be adjusted so that the elastic resistance band(s) is stretched 100% at end of movement. During one visit all exercises will be performed once using one limb at a time, while during the other visit, exercises will be performed using two limbs simultaneously. The six exercises will be performed for a total of three sets with 2 minutes of rest between exercises and one minute of rest between sets. Participants will be instructed to contract their limb with maximal effort at each repetition, executing the maximal number of repetitions that can be performed. Speed of movement will be standardized to one second in the concentric phase and one second in the eccentric phase with the use of a Metronome. The loading in these exercises is determined from 25 RM tests performed during the first visit of the acute phase, executed in accordance to recommended guidelines [41]. The loadings obtained from the 25 RM tests will also be considered start loading for the high-repetitive resistance training regimens in the chronic phase of this protocol.

In the acute phase and during post assessment in the chronic phase the following physiological variables will be measured while executing the latissimus row, chest press, knee extension, shoulder flexion, leg curl and elbow flexion exercises:

1. *Muscle oxygenation*
  Muscle oxygenation will be acquired continuously using a surface electrode from a near-infrared spectroscopy system (NIRS) (OxiplexTS, ISS, Champaign, USA) placed on the skin muscle belly.
2. *Cardiorespiratory response*
  Ventilatory and heart rate responses will be determined using a portable gas analysis system (Oxycon Mobile, Viasys Healthcare, Jaeger, Germany). Minute ventilation (VE) oxygen uptake (VO_2_), carbon dioxide excretion (VCO_2,_), heart rate (HR), respiratory exchange ratio (RER) and pulsed oxygen saturation (SpO_2_) will be monitored. Arterial pressure and cardiac output will be noninvasively measured by a finger photoplethysmograpy device (BMEYE, Nexfin HD, Academic Medical Center, Amsterdam, The Netherlands).
3. *Muscle fatigability*
  Isometric knee extension will be assessed in two ways, first, by measuring the maximum voluntary isometric contraction (MVC), and second, by measuring supramaximal twitch tension (TW) from a series of twitch induced by magnetic stimulation of the femoral nerve (Magstim 200 monopulse Bistim; Magstim Co. Ltd., Whitland, Dyfed, Wales, UK). The occurrence of quadriceps fatigue induced by the exercise regimens will be quantified by measuring the fall in both MVC and TW 15 min after the high-repetitive resistance exercises. Exertional symptoms, that is, perceived dyspnea and muscle fatigue will be quantified with the modified Borg scale.
4. *Exercise stimulus*
  The exercise stimulus will be quantified from the product of the number of repetitions and the training load. The area under the curve of the tension generated with the elastic bands over time of muscle contractions will also be calculated (BioPac Acknowledge software).

#### Muscle adaptation

A needle biopsy of the vastus lateralis, performed as described by Bergström and routinely done in our laboratory will be obtained before and after the 8-week intervention to investigate muscle adaptation to training (key proteins of IGF-1/PI3/AKT pathway, FOXOs, MuRF and MABx/atrogin, fiber-type distribution, enzymatic activities and capillarization).

#### Quality of life and feasibility

Quality of life will be determined with the COPD Assessment test (CAT) [42] and the feasibility of the exercise regimens will be assessed by compliance (number of attended training session) and progression of intensity. The occurrence and severity of any adverse events will be recorded.

### Randomization and blinding

To prevent foreknowledge of treatment assignment and to keep the allocation sequence concealed from the participants and the researchers, group allocation will be done after the completion of baseline and inclusion tests by an individual independent of the recruitment process using a randomly permuted blocks design with a computer random number generator. Randomization will be performed with a 1:1 allocation to the intervention or control group. The allocation sequence will be kept in an opaque, sealed and stapled envelope and will be kept concealed until end of outcome assessment. The envelope will be made impermeable to intense light by using aluminum foil inside and sealed using tamper-proof labels. Furthermore, the participants will be given repeated instructions not to reveal their group allocation to the outcome assessors. In case of failure in keeping the outcome assessor blinded (that is, a patient reveals his/her group allocation), a second trained outcome assessor will be available.

### Statistics

The analysis of primary and secondary outcome measurements will include all randomized patients analyzed by their original group in accordance with intention-to-treat. Within group and between group changes will be analyzed using linear mixed models. In addition, within the main analysis, an on-treatment analysis will be performed. On-treatment is defined as at least 75% compliance, in other words at least 18 training sessions. Mixed models will be used to handle missing outcome data. For comparison of single and two limb exercises student t-tests will be performed. A level of 0.05 will be considered valid for statistical significance. For data management and statistical analysis the IBM Statistical package for Social Science (SPSS) version 21.0 will be used.

### Ethical approval and informed consent

The study has been approved by the Ethical Board of the Institut universitaire de cardiologie et de pneumologie de Québec: CER 21111. All participants will receive brief and comprehensible oral and written information in accordance to the Helsinki declaration [43]. Informed, written consent is obtained from all participants before baseline testing.

## Discussion

The prevention of limb muscle dysfunction in patients with COPD is considered of utmost importance [3]. However, the progressive increase in dyspnea during exercise training that is common in COPD [44] may limit patient’s ability to achieve optimal exercise stimulus since it causes premature exercise limitation [11,12]. The origin of exertional dyspnea has been considered multifactorial [44], involving hyperinflation, increased work of breathing and inadequate energy supply to respiratory and limb muscles as well as intrinsic limb muscle dysfunction [45-48].

High-repetitive single limb resistance training, if compared to a control, may increase limb muscle function and functional exercise capacity in patients with COPD while minimizing the occurrence of limiting exertional symptoms [17,14]. However, no comparison to another exercise regimen has been performed and the intramuscular effects and mechanism of action of this exercise training regimen remain unclear. The proposed trial will give new knowledge to this research area by investigating the mechanisms and effects of single limb exercises in comparison to exercises incorporating a larger amount of muscle mass in patients with COPD and healthy individuals. The use of a single limb approach is novel in patients with COPD, and only a few studies have been published on the topic [13,49,15,17,14]. Most studies have focused on aerobic exercises and in only one study was resistance training the main training modality [17,14]. The results from the proposed trial will provide further insights in the field of single limb training by 1) thoroughly assessing dyspnea and muscle specific responses in both upper and lower limbs during and after high-repetitive resistance training in patients with COPD; 2) investigating mechanisms of improvement behind single upper and lower limb elastic band resistance training; 3) examining acute effects of upper and lower limb single limb elastic band resistance training exercises; and 4) exploring muscle fatigability and the cardiorespiratory demand during such exercises. Acute effects and responses to exercise training have been investigated using comprehensive protocols and equipment that may not be suitable in clinical settings. The present project will assess the acute response to training using elastic bands, which should be widely available [17,14]. Furthermore, since both upper and lower limb muscles are impaired in patients with COPD and since the local effects of exercise training only occur in involved muscle, the incorporation of both upper and lower limbs is of great interest [13,49,15]. One ultimate goal is to learn how to optimize the effects of exercise training and how to customize the interventions in COPD. If, as we hypothesize, single-limb high-repetitive resistance training produces more fatigue and less dyspnea and as a result larger physiological adaptations than exercise involving larger muscle groups, this project may eventually influence how patients with COPD are trained. The results of this project will provide novel information that should be instrumental in individualizing patients’ treatment in a way that could be easily implemented in clinical practice.

### Trial registration

The clinical trial was registered before the enrollment of the first participant. Date of trial registration: 4 November 2014. The first participant was randomized on 10 November 2014. ClinicalTrials.gov identifier: NCT02283580. Trial registration has been based on the WHO registration advisory group minimal registration set [50].

### Trial status

The trial was recruiting participants at the time of submission of this protocol 06 January 2015.

## Additional files

Additional file 1:
**Exercise program single-limb group.** Description of data: Detailed description of exercise program (warm-up, exercises and stretching) for single-limb group including illustrations of start and end positions and muscles involved in each exercise. Information on number of sets, repetitions in each set, rest, speed of movement and execution of exercise movements. Additional file 2:
**Exercise program two-limb group.** Description of data: Detailed description of exercise program (warm-up, exercises and stretching) for two-limb group including illustrations of start and end positions and muscles involved in each exercise. Information on number of sets, repetitions in each set, rest, speed of movement and execution of exercise movements.

## Abbreviations

COPD: Chronic obstructive pulmonary disease
RM: Repetition maximum
UULEX: Unsupported upper limb exercise test
