# Supplementary material for: Muscular and functional effects of partitioning exercising muscle mass in patients with chronic obstructive pulmonary disease - a study protocol for a randomized controlled trial
Source: Trials. 2015 Apr 27;16:194. doi: 10.1186/s13063-015-0698-x (PMC4423627; doi:10.1186/s13063-015-0698-x)
Supplement: Additional file 2: — Exercise program two-limb group. Description of data: Detailed description of exercise program (warm-up, exercises and stretching) for two-limb group including illustrations of start and end positions and muscles involved in each exercise. Information on number of sets, repetitions in each set, rest, speed of movement and execution of exercise movements. [file 13063_2015_698_MOESM2_ESM.pdf]

WARM-UP

Exercise	Illustration (Start – end)	Involved muscle Front & Back	Information	Execution
General warm-up	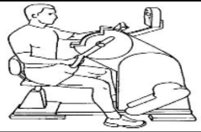		5 minutes using both arms and legs simultaneously.	Use one of the seated machines (NUSTEP or SCI FIT) that incorporates both upper as well as lower limbs. Press quick start. To change resistance press plus or minus. To finish press stop.
Balance	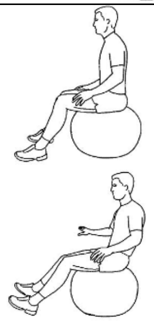	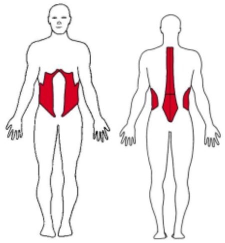	**Time**<br>10 minutes<br>**Set**: 5 set<br>**Repetitions**: 1 minute<br>**Rest**: 1 minute	Sit on the ball, raise your feet from the floor and try to keep your balance. If needed, you may support one hand on a wall to control the direction of movement.

LOW LOAD, HIGH-REPETITIVE RESISTANCE TRAINING

Exercise	Illustration (Start – end)	Involved muscle Front & Back	Information (Set, repetitions, rest and speed)	Execution (Placement of elastic resistance, position and how to perform the exercise)
Rowing	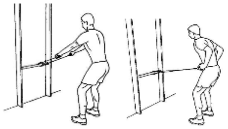	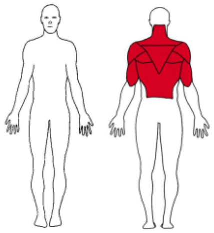	**Set**: 3 set<br>**Repetitions**: Maximal<br>**Rest**: 1 minute<br>**Speed**: 1 second when pulling and 1 second returning arm to start position.	Sit/stand facing the insertion of the elastic band with the arms fully extended in the direction of the elastic band. About 45°. Pull the handle toward your abdomen. Slowly return to the start position and repeat.<br>**Keep in mind:** Use only one arm and use your shoulder blade muscles.
Leg curl	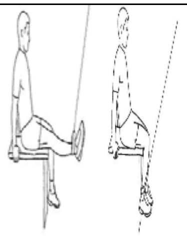	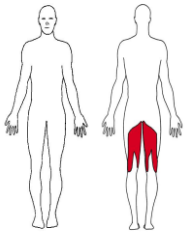	**Set**: 3 set<br>**Repetitions**: Maximal<br>**Rest**: 1 minute<br>**Speed**: 1 second when flexing knee and 1 second returning leg to start position.	Sit facing the insertion for the elastic band. Fasten the elastic band around your ankle. With the exercising leg pointing straight forward, then bend your knee pulling your foot down/inwards under the stool as far as you can. Then release in a controlled manner to the start position with the same speed.<br><br>**Keep in mind:** Flex as much as possible in every repetition

**Exercise**	**Illustration**<br>(Start – end)	**Involved muscle**<br>Front & Back	**Information**<br>(Set, repetitions, rest and speed)	**Execution**<br>(Placement of elastic resistance, position and how to perform the exercise)
Elbow flexion	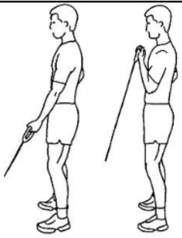	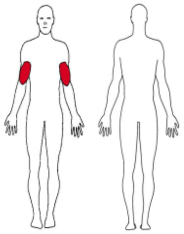	**Set**: 3 set<br>**Repetitions**: Maximal<br>**Rest**: 1 minute<br>**Speed**: 1 second when flexing arm and 1 second returning arm to start position.	Sit/stand facing the insertion for the elastic band with your arms kept straight in the direction of the elastic band. Hold your arms against your body. Move the elastic band upwards by flexing your elbows until the palm of your hands points towards your chest. End position when maximal flexion in elbow joint. Then release in a controlled manner to the start position with the same speed.<br>**Keep in mind:** Fixate your elbows against your body throughout the exercise
Chest press	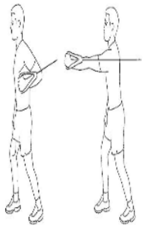	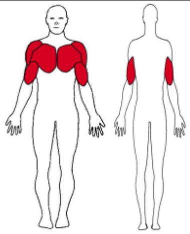	**Set**: 3 set / arm<br>**Repetitions**: Maximal<br>**Rest**: 1 minute<br>(LR rest LR rest LR)<br>**Speed**: 1 second when pushing forward and 1 second returning arm to start position.	Sit/stand with your back facing the insertion for the elastic band and your arm abducted. Your hand should be facing forward with the back of the hand upwards. Press your arm forward until its fully extended, mark your end position and release in a controlled manner your arm back to the start position with the same speed.<br><br>**Keep in mind**: Keep your lower back and abdominal muscles in tension throughout the whole exercise.
Calf	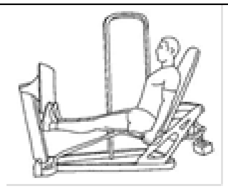	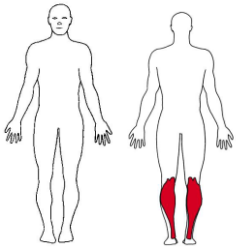	**Set**: 3 set / leg.<br>**Repetitions**: Maximal<br>**Rest**: 1 minute<br>**Speed**: 1 second when extending and 1 second returning leg to start position.	Sit in the chair of the weight machine. Press your feet down and return slowly to the start position. The same movement as when driving a car<br><br>**Keep in mind:** Important that you make a full extension at that your leg is kept straight and that the movement only occurs in the ankle of the foot.
Shoulder flexion	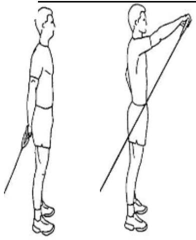	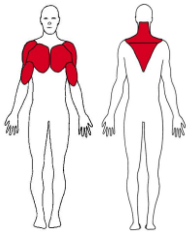	**Set**: 3 set<br>**Repetitions**: Maximal<br>**Rest**: 1 minute<br>**Speed**: 1 second when lifting arm upwards and 1 second returning arm to start position.	Sit/stand with your back facing the insertion for the elastic band. You arms kept straight holding the band with the back of the hand facing upward. Lift your arms upwards/forward until your elbow is in line with your forehead. Then release in a controlled manner to the start position with the same speed.<br><br>**Keep in mind:** Keep your arms fully extended throughout the whole movement

Leg extension	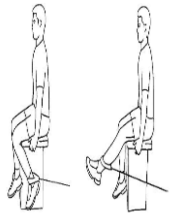	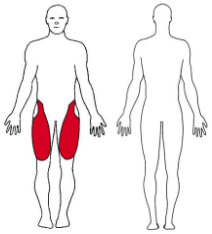	**Set**: 3 set<br>**Repetitions**: Maximal<br>**Rest**: 1 minute<br>**Speed**: 1 second when extending and 1 second returning leg to start position.	Sit with your back facing the insertion for the elastic. Starting position with your knees and hip both in 90°. Fasten the elastic bands around your ankle. Fully extend your legs then release in a controlled manner to the start position with the same speed.<br><br>**Keep in mind:** Fully extend your knee at every repetition
Balance	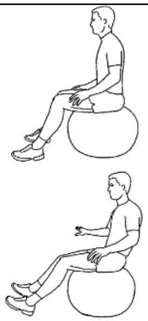	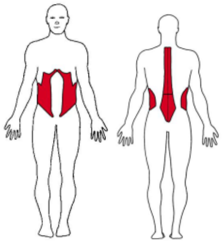	**Time**<br>10 minutes<br>**Set**: 5 set<br>**Repetitions**: 1 minute<br>**Rest**: 1 minute	Sit on the ball, raise your feet from the floor and try to keep your balance. If needed, you may support one hand on a wall to control the direction of movement.

STRETCHING

**Exercise**	**Illustration** (Start – end)	**Involved muscle** Front & Back	**Information** (Set and time)	**Execution**
Back thigh	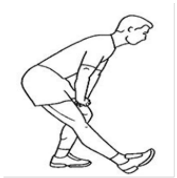	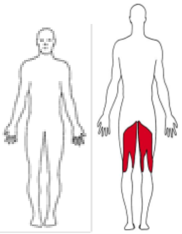	**Set:** 2 set/ leg<br><br>**Time: :** 30 seconds / set<br>Total 2 minutes	Stand with one knee slightly bent and the other leg straight. Support your hands on your knee, keeping your back straight. Slowly lower your upper body forward until you feel the back of your leg stretch. Hold for 30 seconds and change leg. Repeat twice.
Chest	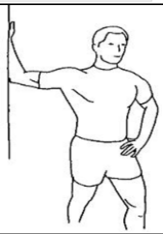	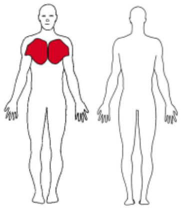	**Set:** 2 set/ arm<br><br>**Time: :** 30 seconds / set<br>Total 2 minutes	Stand with your side to a door frame or something similar. Stretch your arm and place your forearm against the door frame at head height. Slowly turn your body away from the door frame until you feel your chest muscles stretching. Hold for 30 secsonds and change arm. Repeat twice.

Front thigh	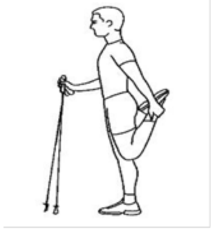	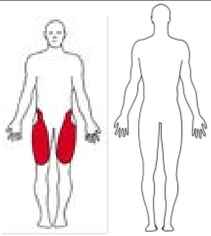	**Set:** 2 set/ leg<br><br>**Time: :** 30 seconds / set<br>Total 2 minutes	Stand with the body straight and support yourself with the rods. Grab one of the ankle, and pull the heel towards the back. Push the pelvic forward, until you feel the front of the thigh stretching. Hold the position for 30 seconds. Repeat with the other leg. Repeat twice.
Calf	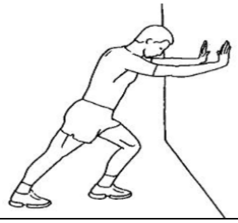	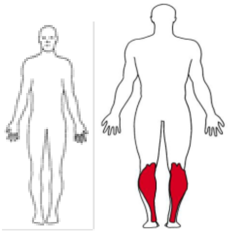	**Set:** 2 set/ leg<br><br>**Time: :** 30 seconds / set<br>Total 2 minutes	Stand inclined facing a wall with one leg bent and the other leg stretched in extension of your body. Keep your heel on the floor and push your hip forward until you feel a stretch in your calf. Hold for 30 seconds and change legs. Repeat twice.
